# In vitro infection of Madin-Darby bovine kidney (MDBK) cells with *Eimeria acervulina* sporozoites: quantitative analysis of parasite cellular invasion and replication using real-time polymerase chain reaction (PCR)

**DOI:** 10.1007/s00436-021-07211-x

**Published:** 2021-06-19

**Authors:** Shahinaz Taha, Tran Nguyen-Ho-Bao, Arwid Daugschies, Zaida Rentería-Solís

**Affiliations:** 1 Institute for Parasitology, Centre for Infectious Diseases, Faculty of Veterinary Medicine, University of Leipzig, An den Tierkliniken 35, 04103 Leipzig, Germany; 2 Department of Preventive Medicine and Veterinary Public Health, Faculty of Veterinary Medicine, University of Khartoum, Shambat, PO Box 32, 13314 Khartoum North, Sudan; 3 Department of Veterinary Medicine, College of Agriculture, Can Tho University, 900000 Can Tho, Vietnam; 4 Albrecht-Daniel-Thaer Institute, An den Tierkliniken 29, 04103 Leipzig, Germany

**Keywords:** Coccidiosis, *Eimeria acervulina*, *Eimeria tenella*, Poultry, MDBK cells

## Abstract

Poultry coccidiosis causes considerable economical losses to the livestock industry. *Eimeria* parasites are responsible for this disease. On a global scale, *E. acervulina* and *E. tenella* are amongst the most common *Eimeria* spp. infecting broilers. *E. tenella* is commonly used as infection model in in vivo and in vitro studies. On the other hand, *E. acervulina* has barely been studied under in vitro conditions. A well established and widely used in vitro model for *E. tenella* infection is the Madin-Darby bovine kidney cell line (MDBK); however, little is known regarding suitability of MDBK cells as host cells for *E. acervulina*. We infected MDBK monolayers with two different doses, 5 × 10^4^ and 2 × 10^5^, of *E. acervulina* sporozoites and evaluated cultures at 24 and 96 h post infection (hpi). For comparison, we ran an identical infection assay using *E. tenella* sporozoites. To assess parasite reproduction, the number of DNA copies of *E. acervulina* SCAR marker and *E. tenella* ITS-1 gene was quantified using real-time quantitative PCR. We found that the number of *E. acervulina* copies increased significantly at 24 hpi in comparison to *E. tenella* (*p* < 0.05). After 96 hpi, *E. acervulina* gene copies were considerably reduced while *E. tenella* continued to multiply (*p* < 0.05). Our results show that MDBK monolayers could be used for in vitro research aimed to study *E. acervulina* sporozoite cell invasion. Nevertheless, modifications of in vitro cultivation appear necessary to allow qualitative and quantitative studies over longer periods of parasite reproduction.

## Introduction

Coccidiosis is an economically important malady in the poultry industry (Blake et al. 2020). The disease is caused by apicomplexan parasites of the *Eimeria* genus. Infection occurs via oral ingestion of sporulated oocysts. Once in the host, the oocysts release sporozoites which invade the intestinal epithelial cells. Inside the host cells, sporozoites undergo asexual and sexual multiplication cycles. Oocysts are thereby produced and consequently shed in the feces. Infected animals can present weight loss, diarrhea, low egg production, and the disease can be fatal in some cases (López-Osorio et al 2020).

Seven species of *Eimeria* (*E. acervulina*, *E. brunetti*, *E. maxima*, *E. mitis*, *E. necatrix*, *E. praecox*, and *E. tenella*) are responsible for avian coccidiosis globally. Oocysts morphology, pathology, and severity of the disease are common differentiating factors amongst these species. From all seven *Eimeria*, *E. acervulina*, *E. tenella*, and *E. maxima* are the most prevalent in broiler farms (Jordan et al 2018; Moraes et al 2015; Györke et al 2013). Out of these 3 species, *E. tenella* is considered highly pathogenic while *E. acervulina* and *E. maxima* show moderate pathogenicity (López-Osorio et al. 2020). Notwithstanding variations in pathogenicity, moderate pathogenic *Eimeria* spp. such as *E. acervulina* could increase the severity of the disease during co-infection (Hiob et al. 2017).

Animal models are a valuable element in infection research. However, in vitro studies of coccidian parasites can contribute to the baseline understanding of the disease on a cellular level (Marugán-Hernández et al. 2020, Bussiere et al. 2018, Thabet et al. 2017). Moreover, they can be a useful tool providing baseline data for future therapies (Thabet et al. 2017; Khalafalla et al. 2011). Due to its ability to grow in non-avian cell lines (Marugán-Hernández et al. 2020; Thabet et al. 2017), *E. tenella* has been widely used as model organism in in vitro research (Marugán-Hernández et al. 2020; Thabet et al. 2019, 2017; Khalafalla et al. 2011) and considerable efforts have been directed to *E. tenella* in vitro and in vivo research, including the use of molecular approaches such as real-time quantitative PCR (RT-qPCR) (Marugán-Hernández et al. 2020; Thabet et al. 2019, 2017; Hiob et al. 2017; Raj et al. 2013). Less efforts have been reported for other *Eimeria* species, including *E. acervulina* (Naciri-Bontemps 1976; Itagaki et al. 1974; Strout et al. 1965; Hiob et al 2017). The aim of this study was to evaluate in vitro invasion and replication of *E. acervulina* sporozoites in MDBK cell monolayers using real-time quantitative PCR.

## Materials and methods

### Passage of *E. acervulina* and *E. tenella* oocysts

Oocysts of *E. acervulina* and *E. tenella* were separately passaged in healthy 11-day-old chicks according to a modified method from Eckert et al. (1995). Sporulated oocysts were collected and stored in 4% potassium dichromate solution at 4 °C until further use.

### Purification and excystation of oocysts

Oocysts of both *Eimeria* species were cleaned from the 4% potassium dichromate solution. Thereafter, sporozoites were excysted and purified according to the modified method described by Rentería-Solís et al. (2020).

### Cell culture

Madin-Darby bovine kidney (MDBK) monolayers (DSMZ, Braunschweig, Germany) were used as infection model. MDBK cells were seeded (2 × 10^5^ cells/well) in 24-well plates with Dulbecco’s Modified Eagle’s Medium (DMEM) supplemented with 10% fetal bovine serum (FBS), 100 IU penicillin, 100 μg/mL streptomycin, and 2.5 μg/mL amphotericin and incubated at 37 °C in an atmosphere of 5% CO_2_ in air until they reached 80% confluency.

### Infection of MDBK cells with *Eimeria* sporozoites

Confluent MDBK monolayers were inoculated with *E. acervulina* sporozoites. Preliminary studies were performed in order to select an infection doses based on different multiplicity of infection (MOI) rates (parasite:cell): 0.25, 0.5, 1.0, 1.5, and 5.0 (Taha et al. unpublished data). Two separate doses were selected for infection: 5 × 10^4^ (MOI: 0.25) or 2 × 10^5^ sporozoites/well (MOI: 0.5). Cultures exposed to infection were then incubated at 41 °C in DMEM medium with 2% FBS, 100 IU penicillin, 100 μg/mL streptomycin, and 2.5 μg/mL amphotericin. Two different incubation times were implemented: 24 h post infection (hpi) and 96 hpi. An identical set of infection doses and incubation times was conducted for *E. tenella* sporozoites. All experiments included one negative control consisting of uninfected MDBK monolayers (NC-uninfected cells). All assays were performed in triplicates. After 24 hpi, cells were washed three times with sterile PBS (pH 7.2) and new medium was added to the 96 hpi group whereas the 24 hpi cultures were terminated. Monolayers were trypsinized at the end of each incubation period (24 hpi or 96 hpi, respectively).

### DNA extraction and real-time quantitative polymerase chain reaction (RT-qPCR)

DNA was extracted from the trypsinized cells using the DNeasy Blood & Tissue kit (Qiagen, Hilden, Germany) according to the manufacturer’s protocol. RT-qPCR was performed to quantify copies of *E. acervulina* sequence characterized amplified region (SCAR) marker Ac-R01-1731 and for *E. tenella* internal transcribed spacer 1 of ribosomal DNA (ITS-1) gene as correlate of parasite replication. RT-qPCR assays were conducted according to the methods described by Blake et al. (2008) and Kawahara et al. (2008), respectively, with some modifications. Briefly, a 20 µl volume reaction contained 10 µl SYBR Green® master mix (Thermo Scientific, Dreieich, Germany), 500 nM of forward and reverse primers (Table 1), 2 µl of DNA template, and 7 µl nuclease-free water. A non-template control (NTC) consisting of nuclease-free water was added to each assay. RT-qPCR reactions were amplified in triplicates and conducted on a Bio-Rad CFX Connect Real-Time PCR Detection System (Bio-Rad, Feldkirchen, Germany). RT-qPCR conditions were 95 °C for 5 min followed by 40 cycles of 95 °C for 30 s. Annealing was performed at 59.8 °C and 58 °C for 20 s, for *E. acervulina* and *E. tenella*, respectively, followed by one extension cycle of 20 s at 72 °C. A melting curve program was applied involving a temperature range from 60 to 95 °C to create a dissociation curve. Finally, *E. acervulina* and *E. tenella* standard curves were generated by a serial dilution of genomic DNA and a serial dilution of cloned ITS-1 gene fragments (according to Thabet et al. 2015), respectively.

**Table 1:** Species-specific set of primers for real-time quantitative PCR

Oligonucleotide identity	Primer sequences (5′ to 3′)	Amplicon size (base pairs)	References
*E. acervulina* forward	Eac_qPCRf CTCGCGTGTCAGCACTACAT	124	Blake et al. (2008)
*E. acervulina* reverse	Eac_qPCRr GATAGCGTGCTTTGCCTTTC		
*E. tenella* forward	Et_qPCRf TGGAGGGGATTATGAGAGGA	147	Kawahara et al. (2008)
*E. tenella* reverse	Et_qPCRr CAAGCAGCATGTAACGGAGA		

### Statistical analysis

D’Agostino-Pearson and Shapiro–Wilk normality tests were used to determine normal distribution of data. A two-way ANOVA test was used for comparison of reproduction considering time points, infection doses, and *Eimeria* species. Differences were considered statistically significant when *p* > 0.05. All statistical analysis were made in GraphPad prism 9 software (San Diego, CA, USA).

## Results and discussion

**Fig. 1:**
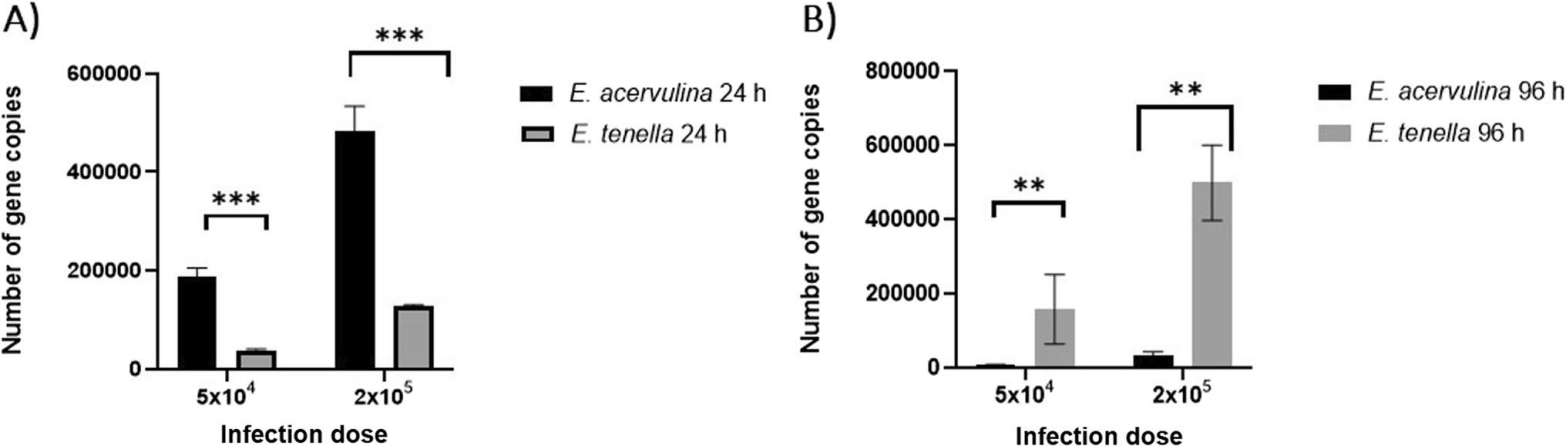
*E. acervulina* and *E. tenella* parasite multiplication. **A** Comparison between *E. acervulina* and *E. tenella* number of copies detected 24 hpi. **B** Comparison between *E. acervulina* and *E. tenella* number of copies detected 96 hpi. **p < 0.005, ***p < 0.0005, infection doses: sporozoites/well

Gene copies of *E. acervulina* SCAR marker and *E. tenella* ITS-1 gene were successfully amplified and detected through RT-qPCR in every reaction. After an incubation period of 24 hpi, the number of copies detected after application of a dose of 5 × 10^4^ sporozoites was significantly higher (*p* = 0.0002) for *E. acervulina* (1.88 × 10^5^ ± 5.56 × 10^4^) than for *E. tenella* (3.60 × 10^4^ ± 5.37 × 10^3^) (Fig. 1). Similarly, a significantly higher (*p* = 0.0002) number of copies were obtained for *E. acervulina* (4.82 × 10^5^ ± 8.50 × 10^4^) than for *E. tenella* (1.27 × 10^5^ ± 9.32 × 10^3^) 24 hpi after application of 2 × 10^5^ sporozoites (Fig. 1).

In contrast, 96 hpi following infection with 5 × 10^4^ sporozoites, the number of copies in the *E. acervulina* infected cultures was significantly (*p* = 0.0044) lower (6.96 × 10^3^ ± 3.87 × 10^3^) as compared to *E. tenella* (1.24 × 10^5^ ± 1.01 × 10^5^). Likewise, the higher dose of 2 × 10^5^ sporozoites incubated with monolayers for the longer period of 96 hpi resulted in significantly (*p* = 0.0044) lower quantities of *E*. *acervulina* copies (3.35 × 10^4^ ± 1.53 × 10^4^) in comparison with *E. tenella* (4.98 × 10^5^ ± 1.28 × 10^5^) (Fig. 1).

We tested the ability of *E. acervulina* to invade MDBK monolayers and to subsequently multiply over 24 and 96 hpi. Earlier attempts to evaluate *E. acervulina* culture have been conducted with variable results. Strout et al. (1965) infected different primary (chicken embryo kidney and fibroblasts) and permanent cell lines (mouse fibroblasts, HeLa cells). Strout et al. (1965) reported recognizable cell infection at 24 hpi in all cell lines. Interestingly, they did not observe parasitic growth over periods of more than 24 hpi in any of the tested cell models (Strout et al. 1965) which is in accordance with our observations of decreasing numbers of gene copies 96 hpi. Naciri-Bontemps (1976) reported growth of *E. acervulina* in chicken kidney cells until 93 hpi and observed oocyst formation after inoculation of merozoites. However, to the best of our knowledge, these findings were not confirmed by later publications.

Only one article has been published on MDBK monolayers infected with *E. acervulina* sporozoites (Talebi 2001). In short, the author exposed MDBK cells previously treated with hyperimmune chicken or rabbit antisera to *E. acervulina* sporozoites for 24 hpi. Cultures were stained and intracellular sporozoites were microscopically counted. Unfortunately, the study solely presents percentages of parasite inhibition by antiserum (Talebi 2001), and thus, conclusions on efficacy of infection in this model cannot be easily drawn. To our knowledge, no further attempts to use MDBK cells as infection model for *E. acervulina* have been reported.

In accordance to the findings of Strout et al. (1965), we observed peak parasite reproduction at 24 hpi and a distinct decrease later represented by low copy numbers at 96 hpi. Strout et al. (1965) and Naciri-Bontemps (1976) reported qualitative data that originated from microscopy analysis only. However, RT-qPCR is a more accurate and sensitive means to assess parasite reproduction. In fact, RT-qPCR is nowadays commonly used for quantitative evaluation and has been established successfully to assess reproduction of coccidia (e.g., Marugán-Hernández et al. 2020, Rentería-Solís et al. 2020, Thabet et al. 2019, Bussiere et al. 2018, Hiob et al. 2017, Thabet et al. 2017, Raj et al. 2013, Khalafalla et al. 2011).

However, electron microscopy could provide valuable data for analysis of intracellular development of *E. acervulina* in MDBK cells. Therefore, it should be considered for further in vitro investigations of *E. acervulina*.

Theoretically, avian cell lines would be preferable as in vitro infection model for chicken *Eimeria* as they originate from the natural host and might allow more representative insight into interactions between parasite and host than mammal-derived cell cultures. However, most of the chicken cell cultures suitable for poultry coccidiosis research are primary lines (Bussiere et al. 2018, Strout et al. 1965; Naciri-Bontemps 1976). Primary cells have several limitations compared to permanent cultures. One is the general need for fresh animal tissue to start laboratory experiments, which may be related to a risk of contamination (Verma et al. 2020). Moreover, to obtain primary cells, animals have to be sacrificed which is in conflict with ethical considerations. Finally, standardization of primary cell lines may be related to difficulties.

Therefore, the use of immortalized cell lines is general practice in most research groups working on in vitro culture of avian coccidia. In general, mammal-derived permanent cultures are chosen, since they are easily available from commercial sources and tools like antibodies, markers, published protocols, genomic sequences, etc. are established, while this is not always the case for less commonly used chicken cell lines.

MDBK cells are a bovine-derived permanent line used as in vitro model for a broad variety of applications. MDBK cells are well established for in vitro studies on *E. tenella* (Marugán-Hernández et al. 2020, Rentería-Solís et al. 2020, Thabet et al. 2019, Bussiere et al. 2018, Thabet et al. 2017, Khalafalla et al. 2011). Marugán-Hernández et al. (2020), for example, conducted a comprehensive description of intracellular development of *E. tenella* in MDBK cells. In this study, the authors tracked via RT-qPCR and reverse-transcriptase real-time PCR cellular division and stage development of transgenic strains of *E. tenella*.

Our goal was to quantify *E. acervulina* multiplication in MDBK cells by PCR technology. To the best of our current knowledge, such data has not been published before. Morphological analysis was not considered in our experiment. However, it has been repeatedly (Marugán-Hernández et al. 2020, Thabet et al. 2017 and Raj et al. 2013) shown that increase of gene copy numbers is in fact related to multiplication during merogony. Development beyond this phase of asexual multiplication is rather unlikely under the conditions given in our experiment.

We found that in comparison to *E. tenella*, *E. acervulina* sporozoites invade the cell and multiply at a higher rate during the first 24 hpi. However, the numbers of gene copies drop considerably by 96 hpi, showing dynamics of parasite multiplication for *E. acervulina* that distinctly differ from those of *E. tenella*. Thus, it appears likely that the various *Eimeria* species behave differently in in vitro culture and that general conclusions obtained from *E. tenella* as the only established model organism should be drawn with caution. The addition of more time points could be helpful to evaluate the dynamics of in vitro multiplication in more detail. Additional techniques can be applied to elucidate what happens during this period and if practical applications for future therapies can derivate from these results.

Nevertheless, we have shown parasite invasion success in this cell line. Therefore, MDBK cells could be further used as infection model for *E. acervulina* sporozoite cell invasion. Also, the use of RT-qPCR and other sensitive tools is recommended. More importantly, this cell line also supports *E. tenella* infection. This could be translated into comparative studies between both *Eimeria* species.

Further detailed quantitative and qualitative analyses should be performed to assess the suitability of MDBK culture as in vitro matrix for *E. acervulina* and developmental stages of other chicken *Eimeria* species.
